# Does Level of Equality in State of Residence Relate to LGBT Health? An Analysis of Adults Aged 50+ Years from 34 US States

**DOI:** 10.1093/geroni/igab046.3171

**Published:** 2021-12-17

**Authors:** Christi Nelson, Ross Andel

**Affiliations:** University of South Florida, Tampa, Florida, United States

## Abstract

Lesbian, gay, bisexual, and transgender (LGBT) health disparities have been well documented in previous research. This study examined whether the level of equality in state of residence (high, medium, fair, poor, or negative), determined by tallied LGBT-related laws and policies, was associated with health outcomes for LGBT adults. This study consisted of 3486 LGB and 959 transgender adults ages 50+ as well as 1:1 propensity matched heterosexual and cisgender participants from the 2018 and 2019 Behavioral Risk Factor Surveillance System (BRFSS) surveys. Separate logistic regression analyses for the LGB, transgender, heterosexual, and cisgender groups were conducted to assess health differences by state equality ranking. Results indicated that LGB participants in fair ranked states were more likely to report fair/poor general health (aOR=1.4, 95% CI=1.1-1.8) and 14 or more days of poor mental health in the past 30 days (aOR=1.4, 95% CI=1.1-1.9) compared to LGB in high ranked states. LGB participants in a low or negative ranked state were more likely to report fair/poor health (aOR=1.6, 95% CI=1.3-2.0), 14 days or more of poor physical health (aOR=1.5, 95% CI=1.1-1.8), and 14 or more days of poor mental health (aOR=1.3, 95% CI=1.0-1.7) in the past 30 days. Transgender participants in medium and low/negative ranked states were more likely to report fair/poor health (lowest aOR=1.75, 95% CI=1.3-2.5) compared to transgender individuals in high equality states. Similar results were not found for the matched heterosexual and cisgender groups. These results suggest that LGBT-related laws and policies may play a role in LGBT health.

